# Impact of Boundary Conditions on the Behavior of Thin-Walled Laminated Angle Column under Uniform Shortening

**DOI:** 10.3390/ma14112732

**Published:** 2021-05-21

**Authors:** Jarosław Gawryluk

**Affiliations:** Department of Applied Mechanics, Lublin University of Technology, Nadbystrzycka 36, 20-618 Lublin, Poland; j.gawryluk@pollub.pl; Tel.: +48-815384144

**Keywords:** flexible pad, laminated angle column, compresion, FEM, experiment

## Abstract

Determining the appropriate boundary conditions of a structure is a very important aspect in the failure analysis. In experimental tests, the method of compressing composite samples significantly influences the obtained results. In numerical studies, there is a problem of correctly defining the boundary conditions applied in real object. Therefore, many numerical tests on samples should be undertaken to observe their behavior and to determine ultimate load. The present work includes study to determine the impact of boundary conditions on the thin-walled laminated angle column under compression. The phenomenon of buckling and the post-buckling bahavior of columns were investigated experimentally and numerically. First, the real simply supported angle columns subjected to uniform shortening are tested. Due to the stress concentration between the real sample and the grips, a flexible pads were used. Experimental tests are carried out on the universal testing machine. The deformations of columns were measured using the non-contact Aramis System. The composite material condition was monitored by acoustic emission using the Vallen Systeme with piezoelectric sensors. Next, the numerical calculations in Abaqus software based on the finite element method are performed to validate the empirical results. To determine the influence of the boundary conditions, two numerical models of the system with and without flexible pads are developed. To estimate damage initiation load in numerical models a different damage criteria ( Tsai-Hill, Tsai-Wu, Azzi-Tsai-Hill, Hashin) are used. Based on the results specified that the model with elastic pads more accurately reflects the actual behavior of the L-profile element under compression. It was supported, i.e., by good agreement of flanges deflection (the equilibrium paths) with experimental results. Furthermore, a qualitative and quantitative agreement of damage initiation load were obtained using Hashin criteria (error 4.61%).

## 1. Introduction

Loss of stability is an important process during operation of thin-walled structures, which can lead to the complete damage of the structure. Therefore, a detailed analysis of the structure failure under the load is extremely necessary from an engineering point of view. In particular, the problem is encountered in the aerospace industry [1,2], where isotropic materials are replaced with modern composite materials, which are characterized by excellent strength parameters with a significant weight reduction of these elements. An important issue is description the damage process of thin-walled laminated structures [3,4]. In thin-walled structures with flat walls made of composite material, the system works after the loss of stability (the system has stable equilibrium paths) [5,6]. This means that apart from knowledge the critical load value, the behaviour of the structure after loss of stability also has an important role. In most cases, the analysis of post-critical states requires taking into account non-linear relationships between displacements and deformations. Nonlinear calculations are usually performed for structures with initialized geometrical imperfections corresponding to the selected buckling mode of the structure, which was determined in the linear stability analysis. This makes it possible to determine the relationship between the load on the structure and the geometrical parameters determining the displacement of its nodes—i.e., we will obtain post-critical equilibrium paths [7]. By carrying out nonlinear studies in a post-critical state, the moment of failure initiation in a composite structure can be determined. This is particularly important as this phenomenon exhausts the load capacity of the structure. It is quite a complicated and ambiguous problem, as the failure model depends on many factors, i.e., the type of load, geometry and size of the analyzed element, mechanical properties of the materials used, preloads and damages. Moreover, the method of producing the actual structure significantly influences the course of the destruction process. As a result, it leads to many failure mechanisms, i.e., matrix/fiber cracking, local buckling of the fibers or the entire laminate layer, and delamination. In order to describe the respective damage mechanism, many failure models have been developed and various failure criteria have been introduced into the literature. The most popular criteria in practical analyses describe the failure of the first layer: the maximum stress criterion, the maximum deformation criterion, the Tsai-Hill criterion, the Tsai-Wu criterion, and the Azzi-Tsai-Hill criterion. The destruction process occurs when at least one criteria is fulfilled [8]. All the above-mentioned criteria provide information that the moment of damage initiation has caused, but they are not able to clearly define which element of the structure was destroyed. For this purpose, the Hashin’s criterion can be used, which determines the destruction of fibers or matrix [9]. Interesting research describing the influence of boundary conditions on the buckling of beams is described in [10]. The Cold-Formed Steel beams with staggered slotted perforations were analyzed. These beams are being used in light gauge steel construction aiming to enhance both the fire and energy performance. However, these web perforations affect the bending capacity. In order to determine this effect, numerical and experimental studies were carried out. It was found that the influence of staggered slotted perforations on local buckling strength of the CFS beams is relatively small (11%).

In the literature, there are a lot of papers describing destruction process of thin-walled structures [11].The analytical methods are used for the post-buckling analysis, in which the thin-walled structure is described with orthotropic material [12]. In order to verify the theoretical models, it was necessary to conduct laboratory tests [13]. One of the experimental methods allowing a description of the damage process of the laminated structure is the acoustic emission method, which enables monitoring of the damage initiation and development of damage up to the complete destruction of the element. Experimental test of thin-walled chanell section profile subjected to compression are described in the paper [14]. Six different configurations of the glass fibers arrangement in the epoxy matrix, considering only symmetrical laminates were investigated. The tests were carried out on a testing machine with the use of an environment for recording acoustic emissions. This applied method made it possible to observe the structure’s behaviour, thanks to which it was possible to estimate the damage initiation load. Similar studies for carbon-epoxy laminate were presented in the work [6]. The authors experimentally tested four variants of laminate fiber arrangement. The AE method was used experimentally. From the conducted experimental studies, it was possible to estimate the loads initiating the damage of structures. Teter et al. [15] presented compression tests of a thin-walled omega profile made of carbon-epoxy laminate. Experimental tests with the use of acoustic emission were considered. Additionally strain gauges were used to record deformations of selected surfaces. This allowed estimating the damage initiation load and post-buckling equilibrium path.The research carried out in the above works has shown that the acoustic emission method is effective in monitoring structural damage. In the latest works devoted to the damage of thin-walled structures, experimental and numerical studies using the finite element method can be found [16]. Nonlinear buckling analyses have been carried out to predict the initial buckling loads of the pultruded glass reinforced plastic wide flange columns under compression in paper [17]. The authors used new criteria for the web-flange junction, because the failure behaviour of this junction is quite different from web or flanges. Numerical results were compared with experimental, and good correlation was obtained. Rozylo et al. [18] described the numerical and experimental tests of thin-walled profiles with an omega cross-section under axial compression. The non-linear range of structures with geometrical imperfection was calculated in the Abaqus environment by the Newton-Raphson method. The Hashin criterion was used to identify the initiation of structure failure. A high agreement of numerical and experimental results was obtained. Detailed research on the post-buckling analysis of thin-walled laminated profiles with a C-section is presented in [19]. Numerical studies in the Ansys environment and experimental studies were carried out. A high agreement of the results was obtained. Samples subjected to axial compression were analyzed. Similar studies on chanell section profiles were presented in [20], where the Abaqus environment was used, and samples were tested under eccentric loading. In the experimental tests, acoustic emission was used to determine the damage initiation. Teter et al. [21] conducted detailed studies of thin-walled angular columns in various configurations of fiber arrangement under uniform compression. The nonlinear research was carried out with the analytical-numerical and finite element method. The influence of geometrical imperfection on the value of the smallest buckling load was determined. The authors proposed the P−Δ intersection method and the $P-\Delta^{n}$ method, thanks to which it was possible to estimate the value of the buckling load with high and small amplitude of the initial deflection with high accuracy. Similar numerical studies were compared with the semi-analytical method (SAM) based on the Byskov-Hutchinson method in [22]. The load-shortening diagrams of the column in the nonlinear range were developed as well as the influence of imperfection on the buckling load were determined.

Taking the above-mentioned into account, it can be said that there are still too few papers with results presenting the behaviour of thin-walled composite structures with L-profile subjected to compressive load. Therefore, it was decided to conduct experimental and numerical tests. This articule is a continuation of previous studies described in paper [23], where the numerical model of the elastic pad was validated and the influence of its parameters on the obtained results was determined. In the present study, the impact of boundary conditions on the thin-walled laminated angle column in post-buckling behavior is analyzed. Therefore, two numerical models with different boundary conditions were compared with the results obtained experimentally. Linear buckling and nonlinear post-buckling analysis were considered to investigate the post-buckling behavior of the beam under uniform compression. In order to estimate damage initiation load in numerical models a different damage criteria were used. Finally, the equilibrium paths for all analysed cases were determined. Based on the results, it was found that the model without elastic pads characterized a much more stiffness then the second one with pads, as shown by: a greater bifurcation load, damage initiation load in all analyzed criteria, lower shortening and less deflection of the column in its center. However, model with flexible pads shows a similar character to the experimental results, i.e., high convergence of the equilibrium paths and a small error in the damage initiation load (Hashin criteria).

## 2. Problem Statement

Thin-walled composite channel-section column with L-profile subject to axial compression were considered. Buckling and post-buckling of such beams were analyzed. Those investigations were mainly aimed at:

- validation of FE model of ideal column with L-profile with results of experimental tests;
- finding advantages and disadvantages of the proposed numerical models of angle column with and without pad;
- checking an influence of the flexible pad on post-buckling behavior.

The thin-walled profiles under consideration were made of a carbon-epoxy unidirectional prepreg tape using autoclaving technique. The thickness of each ply was approx. 0.045 mm. This column consist of 18 layers with following configuration: [60, 0(2), −60(2), 60(3), −60(2), 0(3), −60(2), 0, 60(2)]T. The considered beam was 300 mm long and the width of flanges was 40 mm. The material properties of the beams were determined in tensile and compression tests described in paper [6]. The following material properties (Table 1) Young’s modulus in two orthogonal directions $E_{1}$ and $E_{2}$, Poisson’s ratio $\upsilon_{12}$, and the shear modulus $G_{12}$ were determined. Additionally, the limit properties of the composite, i.e., tensile strength $F_{TU}$ in two orthogonal directions, compression strength $F_{CU}$ in two orthogonal direction and shear strength $F_{SU}$ were determined. These material properties are presented in Table 2.

To eliminate the effect of stress concentration in experimental test, flexible pads between the thin-walled profile and the testing machine handle were used. To check an impact of the flexible pad on post-buckling behaviour of the thin-walled beam under consideration, numerical model with and without pads were investigated.

## 3. Laboratory Setup

**Figure 1:**
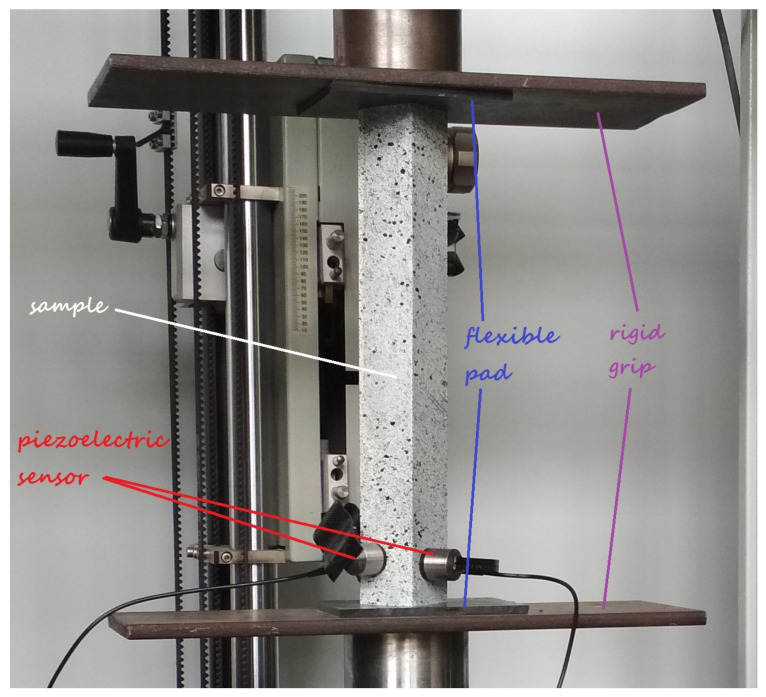
Experimental setup.

Experimental tests consisted in static compression of the fabricated L-profiles on the universal testing machine (Figure 1). In order to eliminate the stress concentration effect between the sample and handle of testing machine, the flexible pads with a thickness of 5.2 mm were used. Laboratory test were performed at a constant velocity of the cross-bar equal to 1 mm/min. The tested columns were loaded with the force value from zero to load, in which damage initiation load was observed. The composite material condition was monitored by acoustic emission. Signals were recorded using the Vallen Systeme, provided with a two piezoelectric sensors. In AE method usually uses the following parameters: the number of hits, the number of counts, the signal amplitude or energy [6,14,15]. In addition, to determine deflection of the sample in the whole range of load, a Aramis system was employed. The ARAMIS is a non-contact measuring system based on digital image correlation doing high-precision measurements with a 3D measurement resolution in the sub-micrometer range, regardless of the specimen’s geometry and temperature. For this purpose, the system used a series of digital photos done at regular intervals during measurements time by two cameras positioned at the appropriate distance from the tested object. I used this system to recording the behavior of the real object, in particular to calculate the shortening of the sample and its flange deflections. The values of the load applied to the system were obtained directly from the testing machine. This allowed to determine a post-buckling equilibrium curves for all tested samples and the value of the load at which the first layer of the laminate was damaged. The experiments were conducted under laboratory conditions at room temperature of 23 ${}^{\circ }$C.

Additionally, verification of the material constants for the flexible pad was carried out in experimental tests. Detailed research was described in paper [23]. However, the obtained substitute material constants are presented in Table 1.

## 4. Numerical Nonlinear Analyses

**Figure 2:**
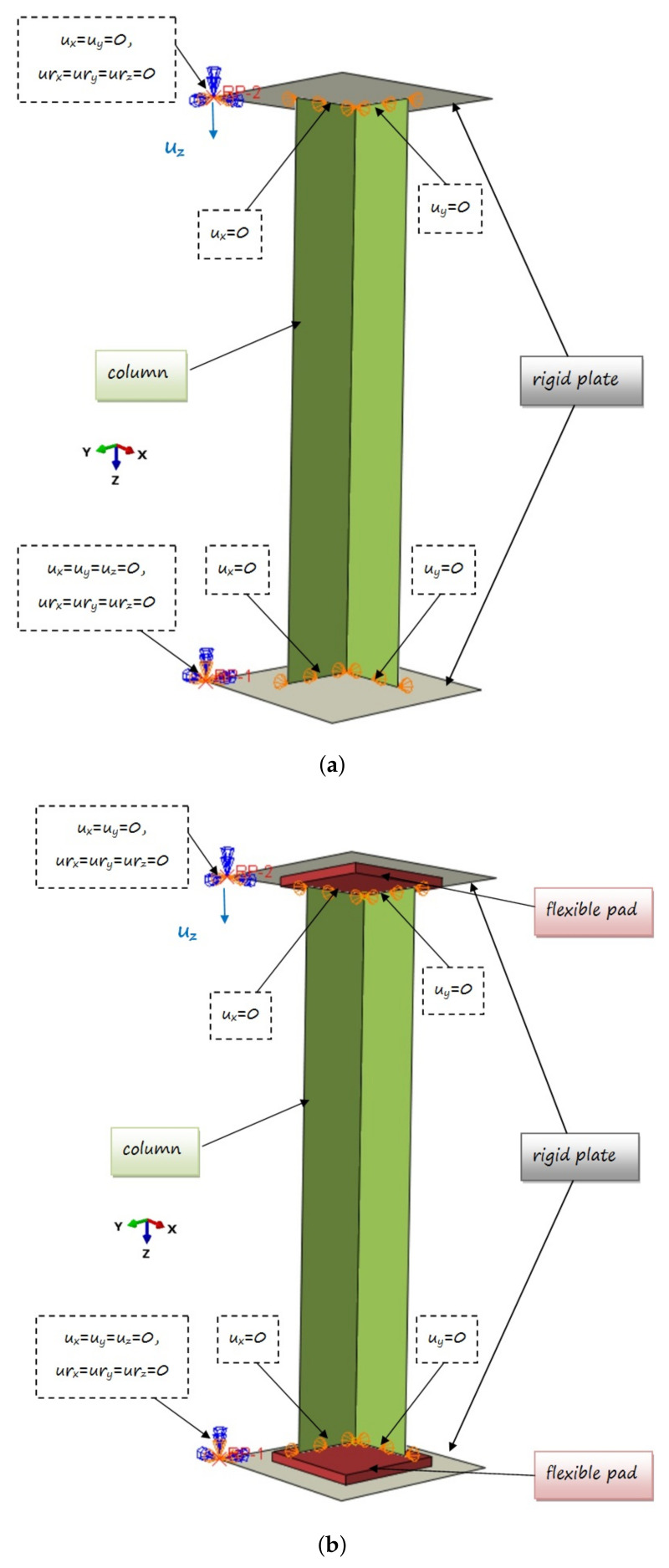
Numerical models of the column: (**a**) M-1; (**b**) M-2.

The numerical nonlinear stability problem was solved with the finite element method (FEM)—ABAQUS software [24]. Two models with different boundary conditions were considered. The first geometrical model (denoted as M-1) consists of only a part of the column situated between the rigid grips (see Figure 2a). The second one (denoted as M-2) has flexible pads between sample and rigid grips are additionally included (Figure 2b). The second assumed model was closer to the structure tested experimentally.

The L-profile laminate column was modelled using S8R shell finite elements. They are 8-node elements with a second order shape function with reduced integration (with six degrees of freedom in each node). The layup-ply technique is used to define the sequence of the laminate layers. Each layer is made of a carbon-epoxy unidirectional prepreg with material parameters presented in the previous section. However, the model of grips are shown as a rigid body (without predefined material properties), made of 4-node, bilinear quadrilateral discrete rigid elements (R3D4) with six degrees of freedom in each node. In the second model (M-2), the flexible pad consists of C3D20R solid element with reduced integration. These are 20-node 2nd order elements (with square shape function) with three translational degrees of freedom at each node. The number of elements was assumed on the basis of the previous experience (e.g., [23]). However, the mesh density was assumed in such a way as not to limit column deformation. The convergence of the model by selecting the size of the elements used (especially the column and pad) was investigated numerically. In the case of the pad, it was determined based on simulations that the number of 3 elements in thickness is sufficient. In both FE models, the thickness of elements discretizing a part of the model corresponding to the considered laminated beam is assumed as 0.81 mm. The system of the L-profile beam with grips was simply supported at two ends. Contact relations were defined between the rigid plate and the profile edge (M-1) or between the flexible pads and the profile edge (M-2) in the normal and tangential directions (friction coefficient set equal to 0.6).

The top rigid plate was locked at the first reference point (RP1), while the bottom rigid plate was allowed to move in the Z direction (at the second reference point (RP2)). The compressive load was realized by displacement of bottom plate along the longitudinal axis of the column. The displacement in the directions perpendicular to the plane of the angle column is set to zero in all nodes located on the profile edges (at the contact points). The specific boundary conditions used in the models are presented in Figure 2.

The above-described numerical models were employed in the numerical calculations to perform an eigenbuckling analysis and a nonlinear buckling analysis to investigate the post-buckling behavior of the beam under consideration. To estimate the moment of the first laminate layer damage, the following initiation criteria were used: maximum stress criterion, Tsai-Hill, Tsai-Wu, Azzi-Tsai-Hill, and Hashin criterions. The value of the damage initiation load and the place where it occurred, according to the above criteria were determined.

## 5. Comparison of Results and Discussion

**Figure 3:**
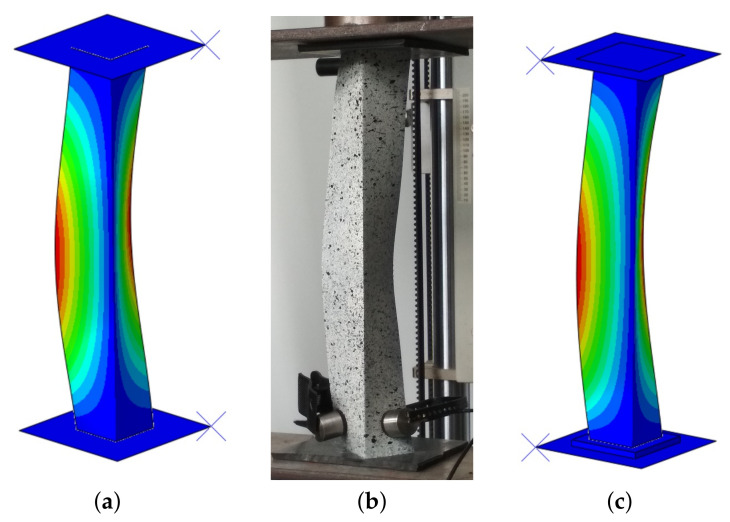
Modes of buckling under axial compression: (**a**) M-1; (**b**) EXP; (**c**) M-2.

The results of the laboratory tests were used to validate the FE models employed to determine the post-buckling behavior of thin-walled structures. The results of the numerical calculations obtained with two models were compared to the results of the experimental investigations. In the linear numerical analysis, the lowest bifurcation load and the corresponding mode of buckling were determined. The same buckling mode was obtained for two models, while the bifurcation load differed by approx 14%. The first model (M-1) obtained a greater bifurcation load, which proves that this model had greater stiffness. However, when the stiffness of flexible pads significantly increased (e.g., E $\times 10^{6}$), the same bifurcation load was obtained in both models. Furthermore, the same mode (along the length of the column one buckling half-wave) in an experimental case was observed (Figure 3).

**Figure 4:**
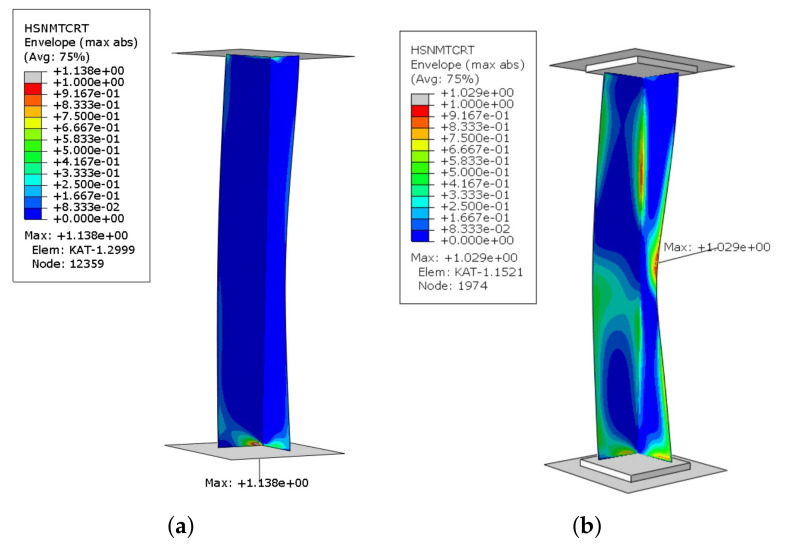
Maps of damage parameter for an angle column: (**a**) M-1; (**b**) M-2.

The maps of the laminate failure parameter for initiation criteria obtained in the numerical calculations shows a different qualitative character in M-1 and M-2 models. Namely, in the first model, due to lack of flexible pads, the damage initiation in all criteria concerns only areas at the profile edges. However, in the second model the damage initiation concerns areas at the profile edges and additionally at the corner of the angle section in 1/2 of its height and in the middle part of one of the flanges. Using the Tsai-Hill, Tsai-Wu, Azzi-Tsai-Hill, and maximum stress criterions, it is possible to catch the moment of damage initiation, but it is not possible to determine the failure mechanism (i.e., what elements of laminate have been damaged). Therefore, the damage initiation criteria based on Hashin theory was used, that allow independent assessment of damage initiation in individual components of the material, i.e., the fibre tensile/compressive initiation criterion (HSNFTCRT/HSNFCCRT) or the matrix tensile/compressive initiation criterion (HSNMTCRT/HSNMCCRT). The initiation of damage occurs when any of the above criteria reaches the critical value of 1. It is used to determine the composite material damage initiation load. In the analyzed sample, the obtained results demonstrate the fulfillment of the Hashin criterion when tensile the matrix (HSNMTCRT). Maps of damage parameter for M-1 and M-2 models are presented in (Figure 4). However, the condition was not achieved in the other failure parameters.

**Figure 5:**
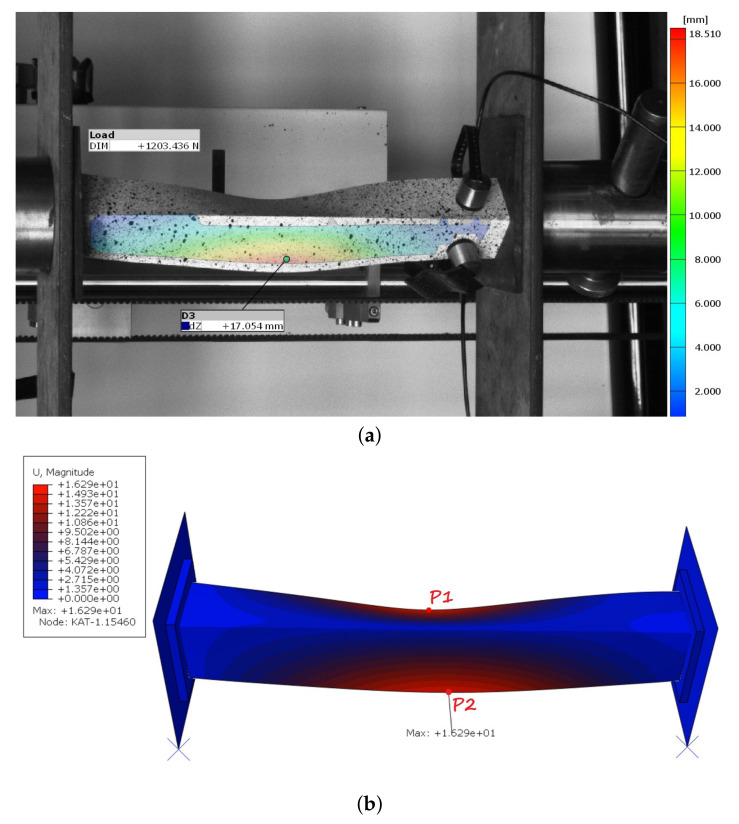
The deflection of an angle column under axial shortening: (**a**) EXP; (**b**) M-2.

The damage initiation in M-2 model occurred mainly in the middle part of one of the flanges, while the place of damage initiation with the Hashin criterion did not change in the first model. Additionally, it was checked in the M-1 model whether the damage mechanism occurring in the M-2 model would appear in further analysis. After completing the necessary research, it was found that the damage mechanism from second model was not observed in the first model. From a practical point of view, the angle section under compression should damage closer to the center than at the edge of the profile. Furthermore, the maximall deflection in the middle of the column was observed in the experimental tests (Figure 5a). It was compared with the second numerical model Figure 5b.

**Figure 6:**
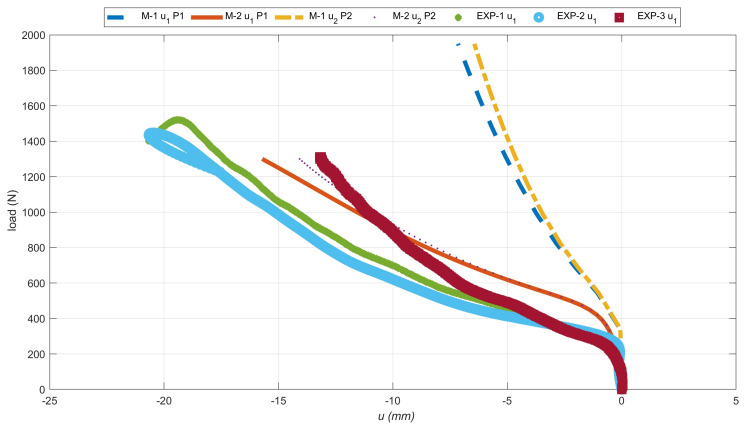
The equilibrium paths, i.e., load-flange deflection relations.

Additionally, the deflection curves of two flanges ($u_{1}$ deflection in the X direction and $u_{2}$ deflection in the Y direction) from two numerical models were compared as a function of the compressive load. Moreover, the above-described curves have been compiled with the deformations recorded by the Aramis system. The deflection of the angle column near the middle, recorded from the experimental and two numerical cases are presented in Figure 6. The deflection of the flange is shown in absolute value, but it should be remembered that not every variant (real sample or numerical model) has a deflection in the same direction. The positioning of the test sample did not allow simultaneous observation of two L-profile column flanges. Therefore, the deflection only one of the sample flanges was recorded.

**Figure 7:**
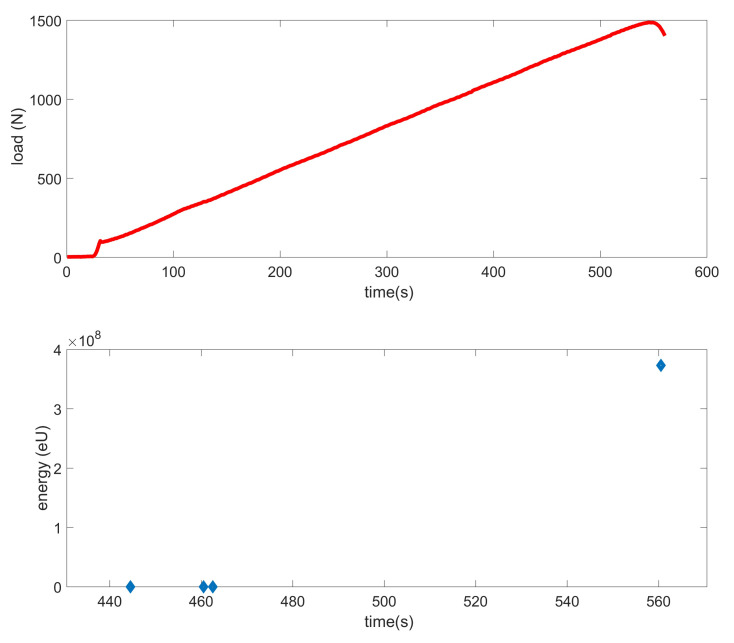
The load and acoustic energy signal versus time for the second real column (EXP-2): Aramis signal—red line; energy signal of AEM—blue mark.

The equilibrium paths clearly show that the first numerical model cis haracterized by a different nature of damage. The deflection in the same node at the damage initiation load differed by about 120%. It relate to both X and Y directions. However, comparing the M-2 model with the experiment, a much smaller error in the range of 11–31% was obtained. Furthermore, the nature of the experimental curves (EXP-1 and EXP-2) complies with that obtained in the second numerical case. In experimental tests, using the AE method, the value of the damage initiation load of the first layer was determined. According to the literature [6,14,15], this type of damage occurs at the first local increase of the acoustic signal energy, which was significantly greater than the previous ones. In Figure 7, such a situation was observed for approx. 560 s, where the energy increased almost eightfold. Thus, the damage initiation load from the load signal at 560s. was assumed. The damage initiation load for other samples in a similar way were determined. Earlier registered lower energy readings could occur as a result of matching the sample to the flexible pads.

**Figure 8:**
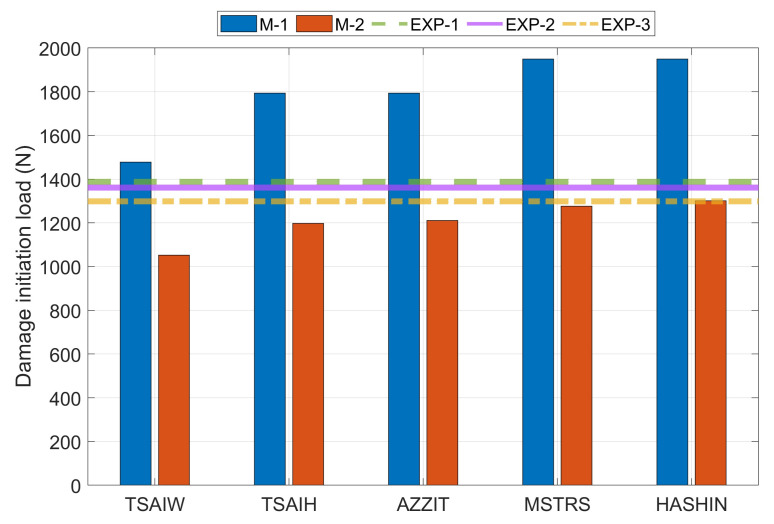
The damage initiation load from numerical and experimental cases.

All the values obtained from the experimental tests were presented in the collective diagram together with the results obtained from the numerical tests (Figure 8),where the following symbols have been adopted: TSAIW—Tsai-Wu criteria, TSAIH—Tsai-Hill’a criteria, AZZIT—Azzi-TsaiHill’a criteria, MSTRS—maximum stress criteria and HASHIN-Hashin’s criteria.

**Figure 9:**
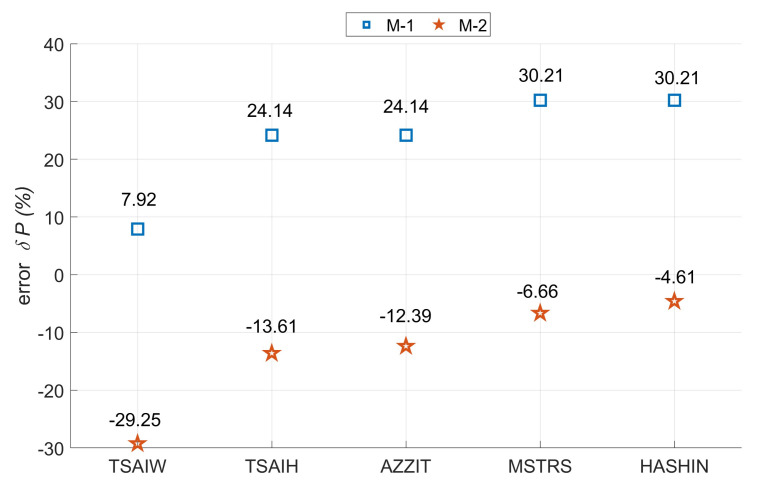
The relative error between numerical and experimental results: $\delta P=(\left(P_{MES}-P_{EXP}\right)/P_{MES})\times 100\;percent$.

The results obtained for the first model were about 28 to 34% higher than for the second one. This confirms the significant influence of the boundary conditions on the behavior of laminated thin-walled columns. Such a large discrepancy in the results may indicate that both numerical models do not analyze the same damage mechanism. Hashin’s criterion on the second model (M-2) almost perfectly defined the damage initiation load compared to the experimental.Based on the three experimental results, the median was determined to be 1361N (EXP-2). Next, relative errors for the numerical results with respect to the median of the experimental results were calculated (Figure 9). The smallest value overestimation was for model M-1 in the case of the Tsai-Wu criterion, while in the other criteria the error increased until it obtained the highest overestimation of the value for the Hashin criteria. However, the smallest underestimation of the value was for the M-2 model in the case of Hashin criterion (4.61%), while in the other criteria the error increased until it obtained the largest underestimation of the value for the Tsai-Wu criterion. In the first case (M-1), the Tsai-Wu criterion allows identifying the damage initiation load, while in the second model, the Hashin criterion definitely helps to identify the damage initiation load. It is worth remembering that in two variants this applies to different damage mechanisms. Considering the above, Hashin’s criterion most accurately describes the actual damage initiation load.

**Figure 10:**
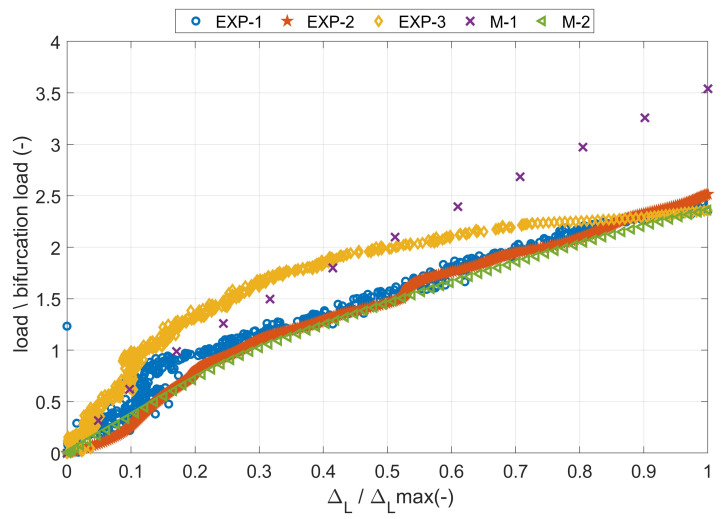
Dimensionless equilibrium paths of L-profile column under shortening.

**Figure 11:**
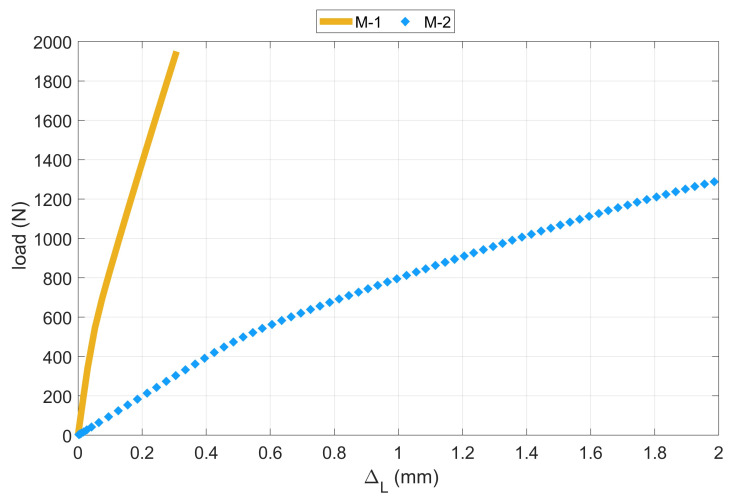
Equilibrium paths of L-profile column under shortening-numerical results.

Dimensionless equilibrium paths determined from experimental and numerical tests are presented in Figure 10. A very good agreement of the results was obtained for the M-2 model, while the results from the M-1 model demonstrate a different damage mechanism. It is also clearly visible in Figure 11, where the equilibrium paths obtained from numerical models are presented. The shortening of the M-1 model was around 550% lower than for the second one. Moreover, the damage initiation load obtained in the first model is higher by about 50% compared to the results of the second model. In addition, it is worth noting how much influence the flexible pad (i.e., the appropriate stiffness of the support) has on the behavior of the thin-walled laminate L-shaped column. It can be suspected that, by increasing the stiffness of the pads, the equilibrium curve should be placed between the presented results.

## 6. Conclusions

The main aim of the study was to validate the numerical models (M-1, M-2) with the results of the experimental tests. The first model M-1 consists of laminated column situated between the rigid grips, while in the second model the flexible pads between sample and rigid grips are additionally included.Based on the obtained results, the following conclusions have been drawn:

- One of the main parameters (the number of half-waves) determining good correlation of the experimental and numerical results is identical.
- The signals from acoustic emissions method should be carefully analyzed, to catch those responsible for damage of the first laminate layer.
- The damage initiation in M-1 and M-2 models shows a different qualitative and quantitative character (Hashin’s criteria), i.e., in the second model occurred mainly in the middle part of one of the flanges, while in the first model it concerns only areas at the profile edges.
- The first model (M-1) was characterized by much more stiffness, as shown by: a greater bifurcation load (about 14%), damage initiation load (up to 34%) in all analyzed criteria, up to 550% lower shortening and less deflection of the column in its center (up to 120%) then the second model M-2.
- The greater flange deflections obtained in real tests may result from inaccuracies in the fabrication of the samples, but they are not easy to identify. For this purpose, a three-dimensional surface analysis should be carried out (using a 3D scanner) and the quality of the actual columns, before the damage test, should be checked.
- The results obtained in the model with flexible pads show a similar character to the experimental results, i.e., the equilibrium paths shows a high convergence and a small error was obtained in the damage initiation load.

Therefore, the model with elastic pads more accurately reflects the actual behavior of the L-profile element under compression. However, further development in numerical models (especially checking more detailed boundary conditions) together with their experimental validation is necessary.

## Figures and Tables

**Table 1:** Properties of used material.

Composite Material	
Young’s modulus $E_{1}$ (MPa)	170,000
Young’s modulus $E_{2}$ (MPa)	7600
shear modulus $G_{12}$ (MPa)	3520
Poisson’s ratio $\upsilon_{12}$ (−)	0.36
**Flexible Pad**	
Young’s modulus (MPa)	40
Poisson’s ratio (−)	0.49

**Table 2:** Limit properties of the composite as determined in compliance with relevant ISO standards.

$F_{\mathrm{TU}}$ (MPa)		$F_{\mathrm{CU}}$ (MPa)		$F_{\mathrm{SU}}$ (MPa)
0${}^{\circ }$	90${}^{\circ }$	0${}^{\circ }$	90${}^{\circ }$	45${}^{\circ }$
1601	14.4	1052	117	90.7

## Data Availability

Data is contained within the article.
